# Gender versus sex: recognizing and adhering to established guidelines for diversity, equity, and inclusion research

**DOI:** 10.1016/j.acpath.2023.100104

**Published:** 2024-02-29

**Authors:** Jeremy W. Jacobs, Garrett S. Booth, Allison P. Wheeler, Deva Sharma, Shannon C. Walker, Brian D. Adkins, Jennifer S. Woo

**Affiliations:** Mayo Clinic, Rochester, MN, USA; Vanderbilt University Medical Center, Nashville, TN, USA; University of Texas Southwestern Medical Center, Dallas, TX, USA; City of Hope National Medical Center, Irvine, CA, USA

**Keywords:** Academic pathology, Diversity, Equity, Gender, Inclusion, Leadership, Sex

We recently read with great interest a study by *Lipscomb* et al. in which the authors examined the rate of advancement from interim to permanent pathology department chair, particularly focusing on gender differences, reasons for serving as interim chairs, and reasons for seeking or not seeking the permanent chair position.^1^ This represents an area of important investigation because, as the authors highlight, despite gender parity among pathology residents and early stage faculty, gender inequities persist among department chairs and senior leadership in academic pathology.

While the study is critical for the advancement of diversity, equity, and inclusion (DEI) in pathology leadership, we wish to highlight an important methodological limitation regarding the appropriate use of the sex and gender terminology in the study. For example, while the authors' intent was to evaluate for potential *gender* differences associated with the advancement of an individual from interim chair to permanent chair, there was consistent use of the *sex* descriptors “male” and “female.” The conflation of gender with sex can be found throughout the article, and the inaccuracies in terminology may be a contributing factor in the survey response rate, as well as a potential etiology for non-responses. Additionally, there is no mention in the materials and methods on how the “male” and “female” descriptors were assigned, though it is assumed the author(s) assigned these attributes, which could lead to inaccurate labeling of a person's identity (e.g., mislabeling of a non-binary individual as a male). Inaccurate characterization of an individual's demographics, including gender identity, hinders one's ability to capture how complex sociocultural factors around sex and gender identity influence rates of academic achievement. In other words, if a researcher mislabels someone's identity as it relates to sex, gender, or both, the researcher has essentially mislabeled all of the social, cultural and political factors that either helped or hindered an individual's opportunities to attain the outcome under investigation—in this study, permanent department chair. Thus, these inconsistencies in methodology potentiate a downstream effect, ultimately hindering our ability to understand how discrete demographic variables influence an individual's promotional and advancement opportunities and outcomes.

While some might potentially counter that this issue is innocuous, numerous national and international organizations, including the American Medical Association (AMA), National Institutes of Health (NIH), Council of Europe (COE), and World Health Organization (WHO) have published guidance on the appropriate usage of sex and gender, and have highlighted the consequences of inaccurate study methods and reporting.^2^, ^3^, ^4^, ^5^ Though intricately linked, sex and gender are discreet variables. For example, the National Academies of Sciences, Engineering, and Medicine describes sex as “a multidimensional biological construct based on anatomy, physiology, genetics, and hormones” with descriptors such as *male*, *female*, and *intersex*; gender refers to “a multidimensional construct that encompasses gender identity and expression, as well as social and cultural expectations about status, characteristics, and behavior as they are associated with certain sex traits” with descriptors including *woman*, *man*, *gender-diverse*, *trans-man*, *non-binary*, etc.^3^ Thus, while frequently used synonymously in casual settings with some overlapping features, sex and gender terms are not interchangeable and should not be conflated, especially within professional literature, as these represent demographic variables with distinct social and health-related implications.^6^

We acknowledge that many databases often conflate sex and gender,^7^ and therefore can impose significant challenges to researchers attempting to study the appropriate or preferred demographic. However, it is paramount that in these instances, researchers carefully and deliberately describe this limitation as a component of their study. This is important, as misappropriation of demographic variables can influence how studies are designed, the accuracy and inclusivity of study results (for example, the inclusion of non-binary and trans individuals), and the interpretation and application of the results. In this study, the method of “gender” assignment was not discussed as a potential study limitation. Likewise, researchers have shown that sex and gender can impact health and disease differently, and may have significantly different societal and policy implications.^8^ Therefore, researchers must be intentional in determining which variable they are studying, assessing, and reporting with accuracy. For example, many gender equity studies have utilized methodology to code or assign “perceived gender” as one cannot know for certain an individual's true gender without querying the individual.^9^, ^10^, ^11^, ^12^, ^13^ However, one may assume an individual's gender based on preferred pronouns and appearance, and utilization of this method must be fully disclosed.

Moreover, while we do not presume the authors’ oversight was intentional, established guidelines and policies for appropriate methods specific to this class of research are freely accessible. These policies are not novel—the AMA guide was published in 2021, and numerous journals have updated their author guidelines in recent years to include policies for appropriate use and reporting of sex and gender.^14^^,^^15^ Indeed, we applaud the journal *Academic Pathology* for having implemented robust author guidelines that explicitly distinguish between, define, and discuss how to report these variables correctly.^16^

Finally, while we commend the authors for their research and their goal of advancing discourse on DEI in pathology, we would like to emphasize that the publication pathway can play a significant role in correcting errors that conflate gender and sex. For this study, several reviewing groups had the potential to correct the terminology and methodology: the study's Association of Pathology Chair's Leadership Development and Diversity Committee, which approved and funded the study; peer reviewers; journal editors; and the publisher and copy editors. This article demonstrates a need for system-wide adjustments to hold ourselves accountable for accuracy. For instance, peer reviewers and journal editors can be guided to review published guidelines on terminology for DEI studies, in particular by publisher and copy editors who presumably have expertise in this area and have access to journal specific guidance.

Implicit biases remain among researchers, as well as across the entire biomedical publishing establishment. Just as submitted journal articles are reviewed for medical content and statistical methodology, we encourage similar scrutiny for DEI principles, ideally via both peer review and in the final editorial assessment prior to publication. This might also include a dedicated DEI editor, a position, which is becoming more common among both peer-reviewed journals and in the journalism and mass-media industry.^17^

In summary, this article advances the discourse on DEI, but also aptly illustrates that we have an opportunity to better educate each other on the richness of human identity, starting with powerful distinction of gender and sex. Accurate reporting of gender and sex has multiple downstream benefits, including broadening the inclusivity of the pathology community, acknowledging the complexity of self-identity in medicine related to biological and sociocultural factors, and first identifying, and then eliminating, barriers to academic advancement with regard to sex and gender. We also must recognize that these demographics, though interconnected, are associated with distinct outcomes for the individual and medical community at large. In the spirit of advancing DEI, we fully support continued efforts to study inequities among different groups, and encourage authors to use language and methodology that best align with current standards.
